# Estrogen Receptor Downregulates Expression of PD-1/PD-L1 and Infiltration of CD8^+^ T Cells by Inhibiting IL-17 Signaling Transduction in Breast Cancer

**DOI:** 10.3389/fonc.2020.582863

**Published:** 2020-09-25

**Authors:** Chong Shuai, Xinmei Yang, Hongming Pan, Weidong Han

**Affiliations:** ^1^Department of Medical Oncology, Sir Run Run Shaw Hospital, College of Medicine, Zhejiang University, Hangzhou, China; ^2^Department of Oncology, The First Affiliated Hospital of Jiaxing University, Jiaxing, China

**Keywords:** immune infiltration, PD-L1, estrogen receptor, Th17 cell, breast cancer, IL-17 family of cytokines

## Abstract

**Background:** The relationship between the interleukin 17 (IL-17) family of cytokines and breast cancer has been widely studied in recent years. Many studies have revealed increased levels of the cytokine IL-17A in estrogen receptor (ER)-negative or triple-negative breast cancer. Upregulation of IL-17A signaling is associated with increased expression of programmed cell death protein 1 (PD-1) and programmed death-ligand 1 (PD-L1) in breast cancer with low ER expression and may elevate the infiltration of CD8^+^ T cells in tumor tissue. This study aims to determine whether ER downregulates the expression of PD-1/PD-L1, reduces the infiltration of CD8^+^ T cells, and affects the immune microenvironment by decreasing T-helper 17 (Th17) cell infiltration and inhibiting IL-17 signaling in breast cancer.

**Methods:** Samples in The Cancer Genome Atlas Breast Cancer dataset were grouped by ER status and the PAM50 intrinsic subtype. The expression of IL-17 family cytokines and Th17 cell signature cytokines were compared between groups. IL-17 signaling pathway-related genes that were differentially expressed according to the ER level were identified. The PD-1 and PD-L1 levels were compared between breast cancer samples with different ER statuses and IL-17A/IL-17F expression levels. Correlation analyses of the expression of PD-1/PD-L1 and IL-17 signaling pathway-related genes were performed. The associations of the expression of IL-17 signaling pathway-related genes with the immune microenvironment were investigated.

**Results:** High levels of ER decreased the expression of IL-17A, IL-17C, and IL-17F but increased the expression of IL-17E (*IL25*), which acts as a suppressor of IL-17 signaling. The expression levels of Th17 cell signature cytokines were significantly increased in ER-negative breast cancer. The expression levels of genes encoding downstream products of IL-17A/IL-17F signaling were downregulated in breast cancer with high ER expression. Increased expression of PD-1/PD-L1 was associated with ER-negative status, IL-17A-positive status, IL-17F-positive status, and upregulation of IL-17 signaling pathway-related genes in breast cancer. Enhanced IL-17 signal transduction was associated with the elevation of CD8^+^ T cell infiltration and variation of the immune microenvironment of breast cancer.

**Conclusion:** High estrogen receptor levels decrease PD-1/PD-L1 expression and CD8^+^ T cell infiltration by suppressing Th17 cell infiltration and IL-17 signal transduction in breast cancer.

## Introduction

Breast cancer (BC) is the most common cancer and the leading cause of death among women worldwide. Estrogen receptor (ER), progesterone receptor (PR), human epidermal growth factor (HER2), and Ki67 levels are the basis for defining the intrinsic molecular subtypes of invasive breast cancer. Previous studies have suggested that ER-negative breast cancer is associated with more frequent distant recurrence, visceral metastasis (1), and poor 5-year overall survival (2). It is currently believed that breast cancer, especially HER2^+^-subtype and triple-negative breast cancer (TNBC), is associated with local inflammation (3). Therefore, biological processes with pro-inflammatory effects, such as the activation of the interleukin 17 (IL-17) signaling pathway, may play a potential role in breast cancer.

The IL-17 family consists of six cytokines that are mainly derived from the CD4^+^ T-helper 17 (Th17) cell subset. IL-17A is the signature cytokine of the Th17 cell subset. IL-17F is highly homologous to IL-17A. IL-17A and IL-17F bind to their corresponding receptors to activate downstream pathways, including the NF-κB, MAPK, and C/EBPs pathways, and then induce the expression of genes encoding several cytokines, chemokines, antibacterial peptides, and matrix metalloproteinases. The IL-17 signaling pathway plays an important role in inflammation, autoimmune diseases, transplant rejection, and tumor and anti-infection immunity. The relationship between the IL-17 family of cytokines and breast cancer has been widely studied in recent years. It has been found *in vitro* and through animal experiments that IL-17A promotes tumor growth (4, 5), metastasis (6), and microangiogenesis (4) through a variety of mechanisms in breast cancer.

Many studies suggest that an increased level of IL-17A in breast cancer tissue is related to ER-negative status or the triple-negative subtype. Cochaud et al. (7) observed increased infiltration of IL-17A-positive cells in ER-negative breast cancer than in ER-positive breast cancer on the basis of immunohistochemistry analysis in 40 breast cancer cases. Zhu et al. (8) confirmed that breast cancer-associated macrophages express IL-17A *in situ* and that IL-17A increases the invasiveness of breast cancer cells *in vitro*. A study of the correlation between the IL-17A level and clinicopathological parameters in patients with breast cancer showed that the number of IL-17A-producing cells in breast cancer tissue is related to a higher histological grade and negative ER/PR status but shows no relationship with tumor stage, tumor size, lymph node status, HER2 status, or histological type (9). Bhat et al. (10) showed that IL-17A expression is increased in ER-negative breast cancer, and increased IL-17A levels are associated with the increased expression of T cell response genes.

The PD-1/PD-L1 axis is considered to be an important target for immunotherapy of breast cancer (11). Increased expression of PD-L1 is observed in inflammatory breast cancers (IBC), ER-negative breast cancer, PR-negative breast cancer, basal tumors, and triple-negative breast cancer (12, 13). Upregulation of PD-L1 is associated with a large tumor size and high tumor grade (13, 14), but it is also associated with improved prognosis in basal tumors or triple-negative breast cancer (12, 13). A study has shown that targeting IL-17A in ER-negative breast cancer inhibits the expression of PD-L1 in tumor cells (15). Therefore, it is supposed that IL-17A is potentially associated with the upregulation of the PD-1/PD-L1 axis in breast cancers with low ER expression.

Immune infiltration in breast cancer is associated with clinical outcome. An increase in the level of CD8^+^ T cell infiltration is associated with ER-negative breast cancer (16). It has been illustrated that tumors lacking immune infiltration are associated with the poorest prognosis in patients with ER-negative breast cancer, whereas an increase in the proportion of CD8^+^ T cells in tumor tissue is associated with improved outcomes in cancers of the same subtype (17). Moreover, a retrospective multicenter study confirmed that increased infiltration of CD8^+^ T cells predicts improved prognosis in triple-negative breast cancer (18) as well as in lymph node-negative breast cancer (19). Considering the function of the IL-17 signaling pathway, the intensity of the IL-17 signaling response is expected to be positively correlated with the infiltration of CD8^+^ T cells in breast cancer, which needs to be proven by analysis.

In this study, bioinformatic analysis based on The Cancer Genome Atlas (TCGA) Breast Cancer dataset was performed to determine whether ER downregulates the expression of PD-1/PD-L1, reduces the infiltration of CD8^+^ T cells, and affects the immune microenvironment by decreasing Th17 cell infiltration and inhibiting IL-17 signaling in breast cancer. First, the association of ER status with the expression of IL-17 family cytokines and signature cytokines in Th17 cells was analyzed. Next, the differentially expressed genes (DEGs) associated with different ER statuses were identified and analyzed among the IL-17 signaling pathway-related genes. Then, the expression of PD-1/PD-L1 was compared between subgroups with different levels of ER expression in breast cancer, and correlation analysis of PD-1 and PD-L1 vs. IL-17 signaling pathway-related genes was performed. Finally, the correlations between IL-17 signaling pathway-related genes and immune cell infiltration in breast cancer, especially infiltration of CD8^+^ T cells, were investigated.

## Materials and Methods

### Analysis of the Association Between the Expression of IL-17 cytokine Family Members and ER Status in Breast Cancer

mRNA expression data for members of the IL-17 family of cytokines (including IL-17A, IL-17B, IL-17C, IL-17D, IL-17E, and IL-17F) and phenotypic information associated with samples of breast cancer were obtained from the TCGA Breast Cancer dataset and analyzed with the UCSC Xena website^1^ (20). ER status was available for 1,046 samples, and the PAM50 intrinsic subtype was available for 781 samples of this dataset. For IL-17A, IL-17C, IL-17E (*IL25*), and IL-17F, positive and negative expression status were defined, respectively, by the presence or absence of the transcript encoding the cytokine. Distributions of the expression status of these cytokines in ER-positive/ER-negative samples were determined to explore the relationship between ER status and cytokine expression levels. For IL-17B and IL-17D, the expression levels of these two cytokines in the ER-positive group and ER-negative group were analyzed and compared quantitatively. Pearson's chi-squared test and Welch's *t*-test were used in this analysis. Tests were performed with Origin software (version 9.1). All tests were two-tailed.

### Analysis of the Effect on Th17 Cell Infiltration of Breast Cancer Tissue According to the Level of ER Expression

The expression data for signature cytokines in Th17 cells, including IL-17A, IL-17F, IL-21, IL-22, and IL-26, were downloaded from TCGA and analyzed online by using the UCSC Xena website. IL-21, IL-22, and IL-26 were qualitatively analyzed by methods similar to those used for the analyses of IL-17A and IL-17F. Moreover, the correlations between signature cytokines and tumor purity in breast cancer samples were analyzed by using the Tumor Immune Estimation Resource (TIMER) website^2^ (21, 22).

### Analysis of the Expression of IL-17 Signaling Pathway-Related Genes in Breast Cancer According to ER Status and the PAM50 Intrinsic Subtypes

A list of 94 IL-17 signaling pathway-related genes was obtained from the Kyoto Encyclopedia of Genes and Genomes database^3^ (ID: HSA04657), and gene expression RNAseq data for these genes were downloaded from the TCGA Breast Cancer dataset by using the R package “TCGAbiolinks” (version 2.12.6). Differential expression analysis was performed by using R package “limma” (version 3.42.2) to assess two expression patterns: (a) a comparison between ER-positive and ER-negative samples and (b) a comparison between the luminal subgroup (including luminal A and B) and the basal-like subgroup. The |log_2_FC| and p value cut-offs were set to 1 and 0.01, respectively.

### Analysis of PD-1/PD-L1 Expression Levels Regulated by ER Status and IL-17 Signaling Pathway-Related Genes in Breast Cancer

mRNA expression data for 94 IL-17 signaling pathway-related genes, PD-1 (*PDCD1*), and PD-L1 (*CD274*) were downloaded from TCGA Breast Cancer dataset. All samples were grouped according to four statuses: (a) ER-positive or ER-negative, (b) PAM50 intrinsic subtype, (c) IL-17A-positive, or IL-17A-negative, and (d) IL-17F-positive or IL-17F-negative. PD-1 and PD-L1 expression levels were compared between groups.

In addition, three cohorts were defined for correlation analyses of PD-1/PD-L1 expression: (a) all available tumor samples in TCGA Breast Cancer dataset; (b) ER-negative samples in TCGA Breast Cancer dataset; and (c) basal-like (triple-negative breast cancer) samples in TCGA Breast Cancer dataset. Pearson's correlation coefficient for the expression correlations between PD-1/PD-L1 and IL-17 signaling pathway-related genes was determined by using R package “stats” (version 3.6.0). Gene pairs for which |Cor.| > 0.25 and *p* < 0.05 were considered significantly correlated gene pairs. The correlations of PD-1 vs. PD-L1 in each cohort were also analyzed as a reference.

### Analysis of the Association of the Expression of IL-17 Signaling Pathway-Related Genes With the Infiltration of CD8^+^ T Cells and the Immune Microenvironment in Breast Cancer

The correlations of the expression of IL-17 signaling pathway-related genes with the infiltration of CD8^+^ T cells and the immune microenvironment in tumor tissues were investigated by using the TIMER website in (a) all tumor samples in TCGA Breast Cancer dataset, and (b) all tumor samples with a basal-like subtype in TCGA Breast Cancer dataset. Among the 94 IL-17 signaling pathway-related genes, expression data for 91 genes was available. A purity-corrected partial Spearman's correlation coefficient was used for data analysis. The immune microenvironment includes the infiltration level of CD4^+^ T cells, CD8^+^ T cells, B cells, macrophages, dendritic cells, and neutrophils.

## Results

### ER Status Is Associated With the Expression of IL-17 Family cytokines in Breast Cancer

**Figure 1:**
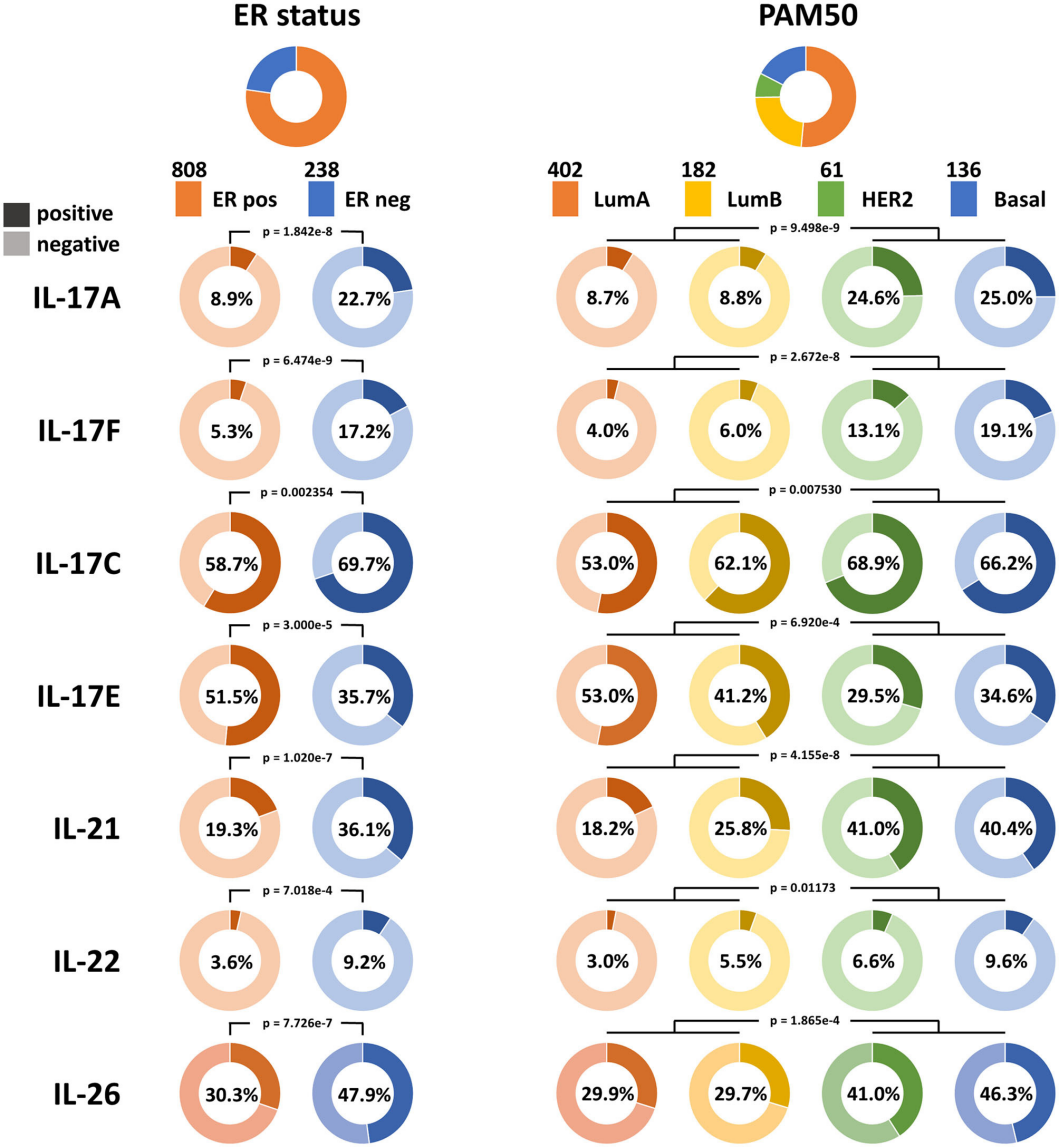
Qualitative analysis of the associations between the expression of IL-17A, IL-17F, IL-17C, IL-17E, IL-21, IL-22, and IL-26 and ER status in breast cancer. The proportions of samples according to ER status and the PAM50 intrinsic subtypes in TCGA Breast Cancer dataset are shown at the top, and the color and sample size of each subtype are indicated. The expression statuses of the six cytokines in each subtype are displayed. A dark color indicates positive expression, a light color indicates negative expression, and the percentage on each ring indicates the positive expression rate of the cytokine in each subgroup.

Bhat et al. (10) reported that the overall expression level of IL-17A in breast cancer is very low. It was also noticed in our study that transcripts encoding IL-17A and IL-17F in the majority of samples and transcripts encoding IL-17C and IL-17E in half of the samples were absent according to TCGA Breast Cancer dataset, which made the quantitative analysis difficult. Therefore, the positive and negative statuses for the expression of these cytokines were defined for qualitative analysis. The distributions of IL-17A, IL-17F, IL-17C, and IL-17E expression are shown in Figure 1. IL-17A is the hallmark cytokine of the Th17 cell subset. IL-17F shares the greatest sequence similarity with IL-17A, is derived from similar cells as IL-17A, signals through the same receptor complex as IL-17A, and often interacts with IL-17A to form heterodimers (23). IL-17F is also considered an inflammatory cytokine that induces the expression of many proinflammatory cytokines and chemokines and is similar to IL-17A (24). Thus, we focused on the expression levels of IL-17A and IL-17F in different subtypes. The positive expression of IL-17A in ER-negative samples was higher than that in ER-positive samples (22.7 vs. 8.9%, RR = 2.55, and *p* = 1.842e−8). The expression of IL-17F was also higher in ER-negative samples than in ER-positive samples (17.2 vs. 5.3%, RR = 2.23, and *p* = 6.474e−9). In addition, the proportions of both IL-17A-positive samples and IL-17F-positive samples were larger for the two nonluminal subtypes (HER2^+^ and basal-like) than for the two luminal subtypes. The consequence indicates that increased expression of IL-17A and IL-17F is associated with low levels of ER in breast cancer.

IL-17C is similar to IL-17A in promoting the expression of inflammatory cytokines and antibacterial peptides, whereas it is mainly derived from epithelial cells (23), which may explain why more positive samples with IL-17C expression were observed than those with IL-17A expression in breast cancer. IL-17C has a similar tendency in its expression distribution as that of IL-17A and IL-17F, which means it has an increased positive rate in ER-negative samples (69.7 vs. 58.7%, RR = 1.19, and *p* = 2.354e−3) and nonluminal samples.

IL-17E induces T-helper 2 (Th2) cell responses by promoting the secretion of Th2-related cytokines (IL-4, IL-5, and IL-13), whereas it suppresses Th17 responses and autoimmune inflammation (25). It was observed that an increase in the proportion of IL-17E-positive samples is related to ER-positive status (51.5 vs. 35.7%, *p* = 3.000e−5) and luminal status, which is in contrast to that observed for IL-17A, IL-17F, and IL-17C. This demonstrates that ER may participate in the downregulation of the IL-17 signaling pathway in breast cancer.

**Figure 2:**
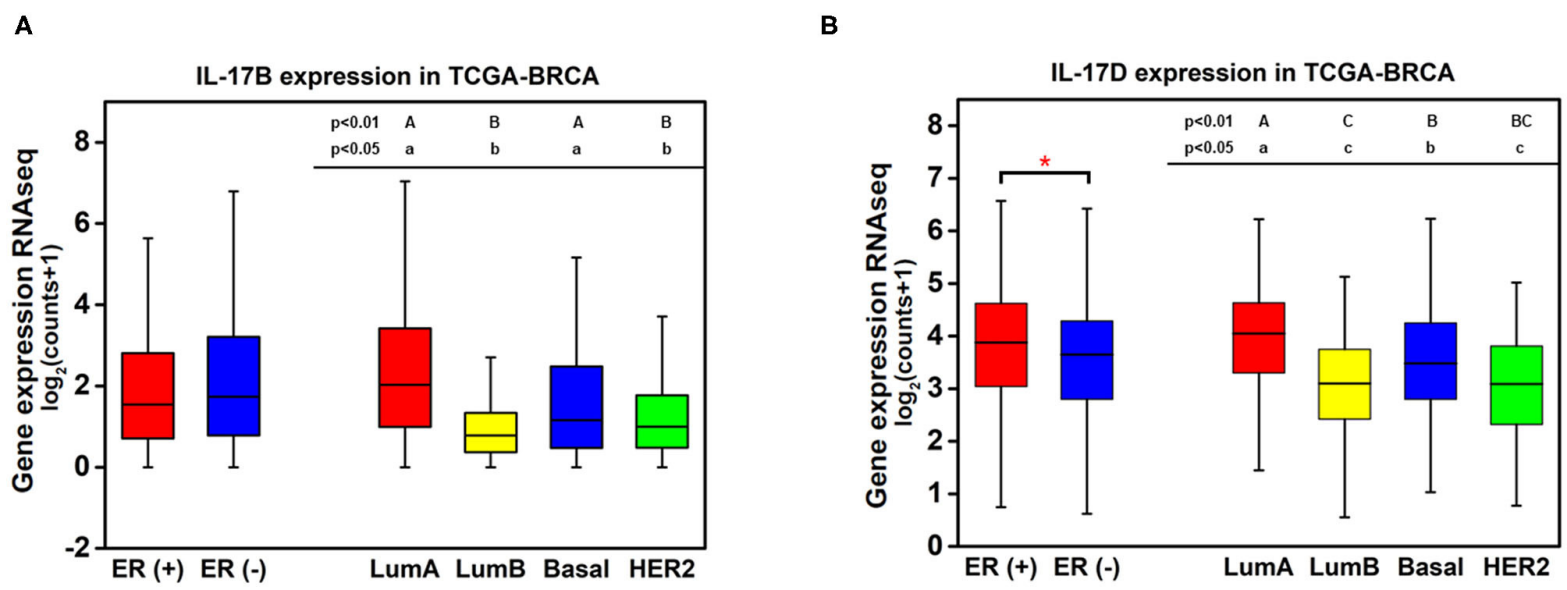
Quantitative analysis of the associations between the expression of IL-17B and IL-17D and ER status in breast cancer. **(A)** IL-17B expression level in breast cancer samples grouped by ER status and PAM50 intrinsic subtypes; **(B)** IL-17D expression level in breast cancer samples grouped by ER status and PAM50 intrinsic subtypes. Welch's *t*-test, *p < 0.05. Letters indicate the statistical significance of multiple pairwise comparisons.

Knowledge of the biological function and signaling transduction pathways of IL-17B and IL-17D remains largely elusive. The expression levels of IL-17B and IL-17D in breast cancer were quantitatively analyzed because they showed much higher expression than other cytokines from the IL-17 family. No significant difference in IL-17B expression between ER-positive samples and ER-negative samples was observed, whereas the expression levels of IL-17B/D in luminal A samples were significantly higher than those in luminal B samples despite their consistency in terms of ER status (Figure 2). This result indicated that ER status may not be the main factor affecting the expression of IL-17B and IL-17D.

### ER Decreases the Infiltration of Th17 Cells in Breast Cancer Samples

**Figure 3:**
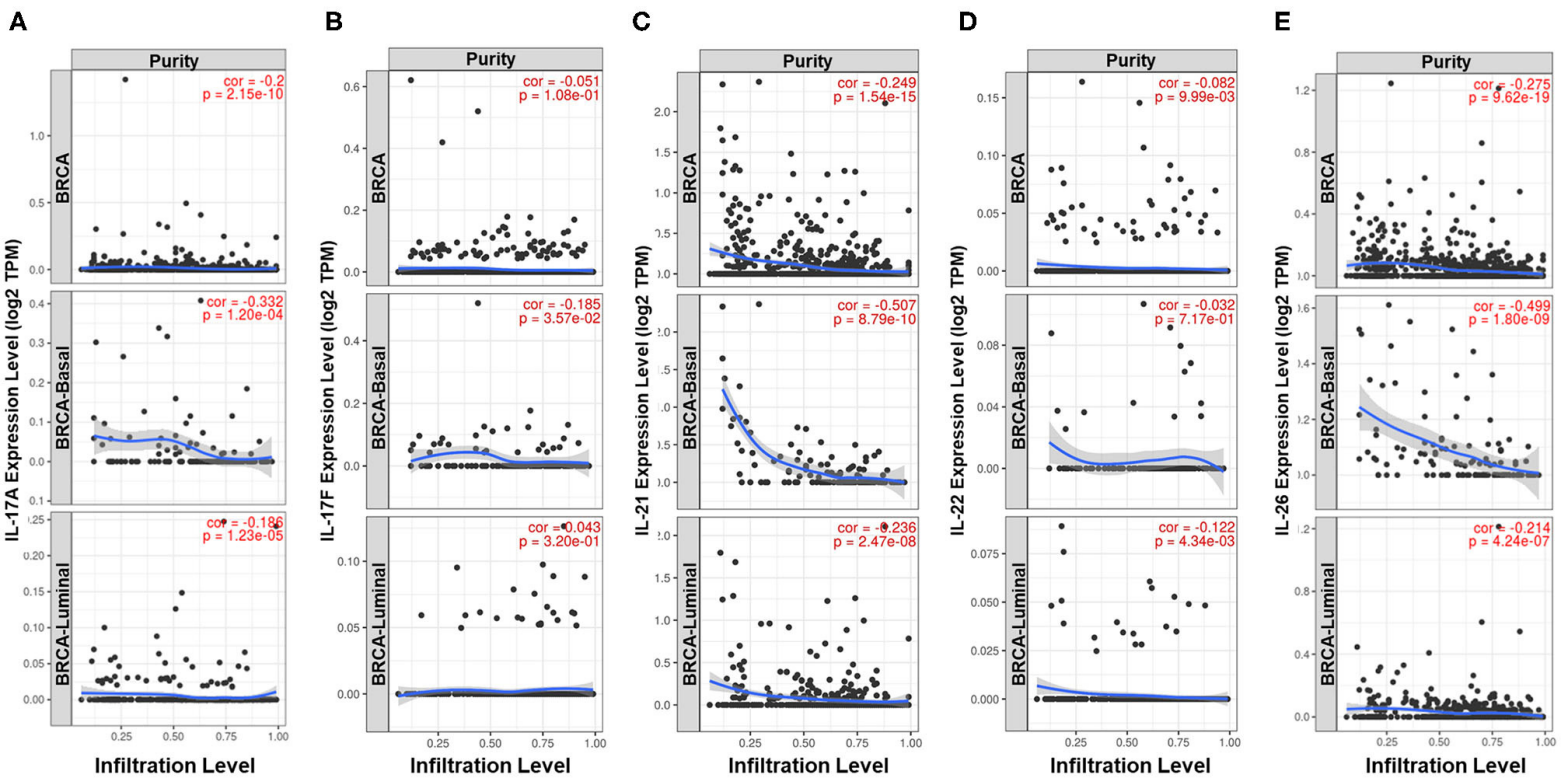
Analysis of the cell sources of five Th17 signature cytokines in breast cancer. Correlation between the expression level of **(A)** IL-17A, **(B)** IL-17F, **(C)** IL-21, **(D)** IL-22, and **(E)** IL-26 and tumor purity in all samples, basal-like samples and luminal samples of TCGA Breast Cancer dataset. The partial Spearman's rho value and statistical significance are shown in red in each panel. The *X*-axis shows tumor purity, and the *Y*-axis shows gene expression.

Previous studies have demonstrated that Th17 cells express a variety of cytokines, including IL-17A, IL-17F, IL-21, IL-22, IL-17E (IL-25), IL-26, GM-CSF, and TNF (26–28). The expression pattern and biological function of IL-17E are the opposite of those of IL-17A, GM-CSF, and TNF and are not specific to Th17 cells; thus, IL-17A, IL-17F, IL-21, IL-22, and IL-26 were defined as signature cytokines for representing the infiltration level of Th17 cells in breast cancer tissue. Qualitative analysis showed a higher positive rate of IL-21, IL-22, and IL-26 expression in ER-negative samples (IL-21: 36.1 vs. 19.3%, *p* = 1.020e−7; IL-22: 9.24 vs. 3.59%, *p* = 7.018e−4; IL-26: 47.9 vs. 30.3%, *p* = 7.726e−7) and nonluminal samples, which is similar to that observed for IL-17A and IL-17F (Figure 1). In addition, the expression of the five signature cytokines is negatively correlated with tumor purity in breast cancer, especially for IL-17A, IL-21, and IL-26 (Figure 3), which indicates that these cytokines are mainly derived from immune cells such as Th17 cells rather than tumor cells. The consequence suggests that ER decreases the infiltration of Th17 cells in breast cancer tissue, which may be the reason for the decrease in IL-17 cytokines in samples with low ER expression.

### Expression of Downstream Products Induced by IL-17A/IL-17F Signaling Is Downregulated by the Estrogen Receptor in Breast Cancer

**Figure 4:**
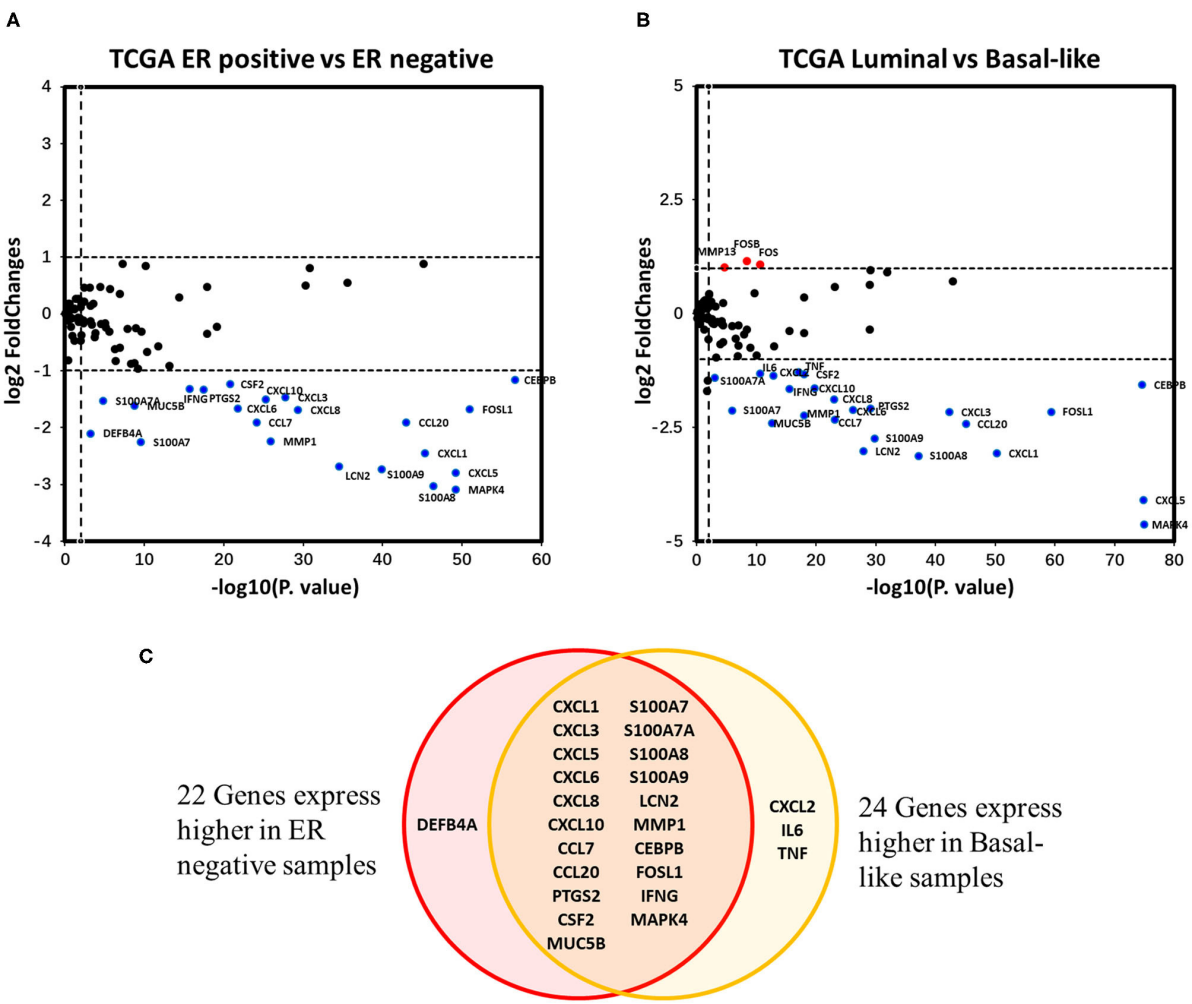
Analysis of the associations between IL-17 signaling pathway-related genes and ER status in breast cancer. **(A)** the Volcano plot shows IL-17 signaling pathway-related genes that were differentially expressed between ER-positive vs. ER-negative samples. The |log_2_FC| and *p*-value cut-offs of significance are 1 and 0.01, respectively. Red indicates genes expressed at significantly higher levels in ER-positive samples, and blue indicates genes expressed at significantly higher levels in ER-negative samples. **(B)** the Volcano plot shows the IL-17 signaling pathway-related genes that were differentially expressed between luminal (both luminal A and B) samples vs. basal-like samples. The |log_2_FC| and *p*-value cut-offs of significance were 1 and 0.01, respectively. Red indicates genes expressed at significantly higher levels in luminal samples, and blue indicates genes expressed at significantly higher levels in basal-like samples. **(C)** Venn diagram showing genes expressed at higher levels in both ER-negative and basal-like samples.

Differential expression analysis of the 94 IL-17 signaling pathway-related genes in breast cancer was performed. Twenty-two genes were identified as DEGs associated with ER status in breast cancer, and all of them were expressed at higher levels in ER-negative samples (Figure 4A). To eliminate the confounding influence of HER2 status, differential expression analysis between the luminal subtype (including luminal A and B) and the basal-like subtype was performed. In the supplementary analysis, among the 27 DEGs identified, 24 genes were more highly expressed in basal-like samples, whereas only three genes were more highly expressed in luminal samples (Figure 4B). Twenty-one genes were expressed at higher levels in both ER-negative samples and basal-like samples (Figure 4C). Interestingly, almost all genes that encode downstream products of IL-17A/IL-17F signal transduction were observed to have higher expression in ER-negative or basal-like samples. These genes include nine genes encoding chemokines (*CXCL1, CXCL2, CXCL3, CXCL5, CXCL6, CXCL8, CXCL10, CCL7*, and *CCL20*), four genes encoding cytokines (*IL6, TNF, CSF2*, and *PTGS2*), seven genes encoding antimicrobial peptides (*MUC5B, S100A7, S100A7A, S100A8, S100A9, LCN2*, and *DEFB4A*), and one gene encoding a member of the matrix metalloproteinase family that functions in tissue remodeling (*MMP1*). This suggests that ER downregulates the intensity of the response to IL-17A/IL-17F signaling in breast cancer, which might result from reduced IL-17A/IL-17F levels and decreased Th17 cell infiltration. In addition, *IFNG, FOS, FOSB1*, and *FOSL1* were also identified as DEGs in our analysis. *IFNG* and *FOSL1* expression were higher in ER-negative or basal-like samples, whereas *FOS* and *FOSB* expression were higher in luminal samples.

### ER-Negative Status and Increased Expression Levels of IL-17 Signaling Pathway-Related Genes Increase the Expression of PD-1 and PD-L1 in Breast Cancer

**Figure 5:**
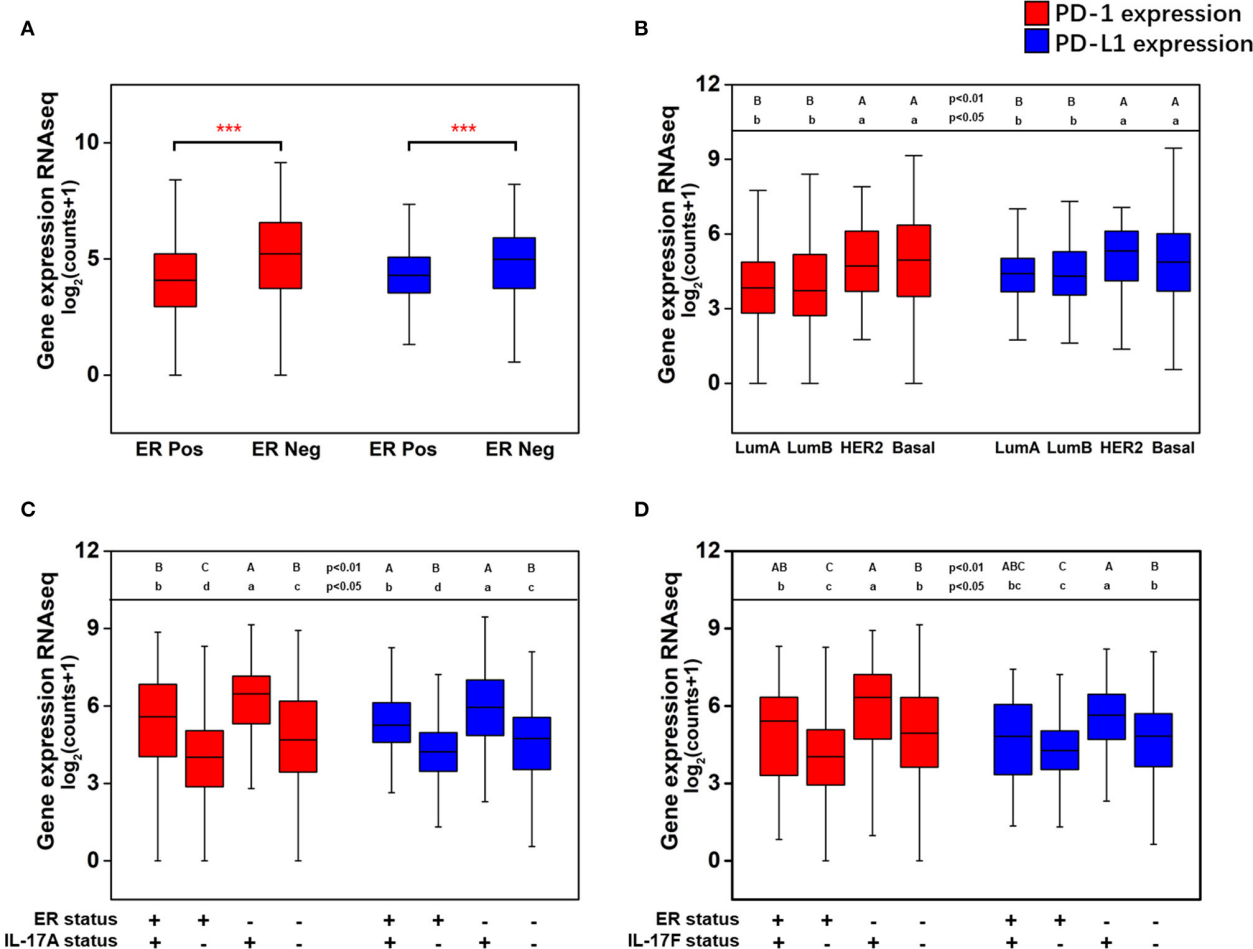
Analysis of the associations between the expression of PD-1 and PD-L1 and ER status, IL-17A expression, and IL-17F expression in breast cancer. **(A)** PD-1 and PD-L1 expression levels in breast cancer samples grouped by ER status; **(B)** PD-1 and PD-L1 expression levels in breast cancer samples grouped by the PAM50 molecular subtypes; **(C)** PD-1 and PD-L1 expression levels in breast cancer samples with different combinations of ER status and IL-17A expression status; **(D)** PD-1 and PD-L1 expression levels in breast cancer samples with different combinations of ER status and IL-17F expression status. Welch's *t*-test, ***p < 0.001. Letters indicate the statistical significance of multiple pairwise comparisons.

The effect of the level of ER expression and the IL-17A/IL-17F expression status on the expression level of PD-1/PD-L1 in breast cancer is presented in Figure 5. The expression levels of PD-1 (median 5.22 vs. 4.07, *p* < 0.001) and PD-L1 (median 4.98 vs. 4.29, *p* < 0.001) were significantly higher in ER-negative samples than those in ER-positive samples (Figure 5A). Moreover, higher expression of PD-1 and PD-L1 were discovered in two nonluminal subgroups, while no significant difference in PD-1 or PD-L1 expression was observed between the basal-like subgroup and the HER2^+^ subgroup or between the luminal A subgroup and the luminal B subgroup (Figure 5B). This suggests that ER status is a critical factor that affects the levels of PD-1/PD-L1 in breast cancer tissues, whereas the HER2 status or Ki67 expression level do not seem to affect changes in the expression of PD-1/PD-L1. In addition, the expression of PD-1 and PD-L1 with different combinations of ER status and IL-17A/IL-17F expression status was compared. It was found that PD-1 and PD-L1 have the highest expression in ER(–)IL-17A(+) and ER(–)IL-17F(+) group (Figures 5C,D). Changes in the expression level of PD-1/PD-L1 due to differences in IL-17A/IL-17F status were more obvious than those due to differences in ER status. This confirms that high expression of PD-1/PD-L1 is correlated with ER-negative and IL-17A/IL-17F-positive status.

**Figure 6:**
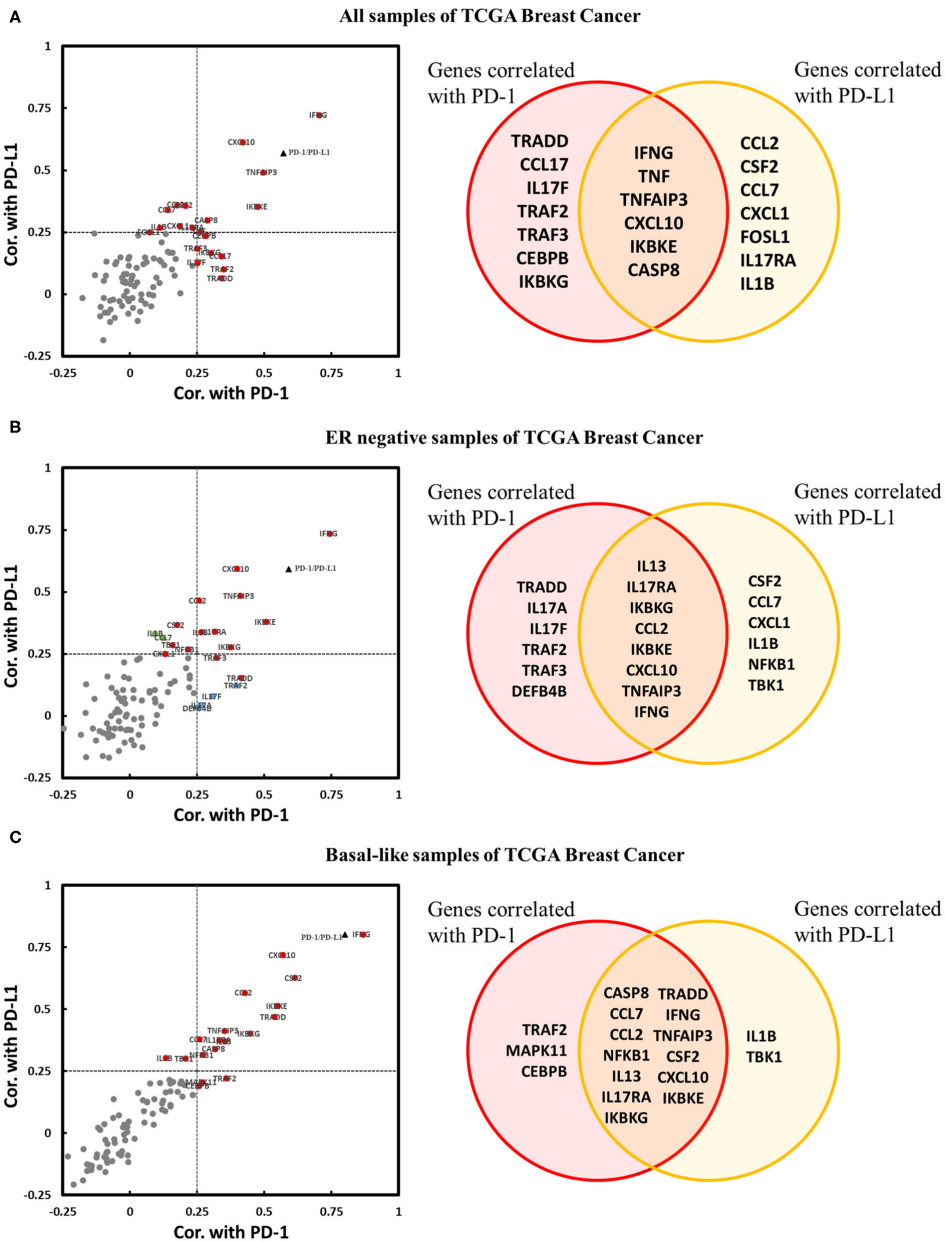
Expression correlation analysis between IL-17 signaling pathway-related genes and PD-1 and PD-L1 in breast cancer. Correlations between IL-17 signaling pathway-related genes and PD-1 and PD-L1 in panel **(A)** all samples in TCGA Breast Cancer, **(B)** ER-negative samples in TCGA Breast Cancer, and **(C)** basal-like samples in TCGA Breast Cancer, as displayed by a scatter diagram and a Venn diagram. The *X*-axis indicates the correlation coefficient with PD-1, and the *Y*-axis indicates the correlation coefficient with PD-L1. Red indicates genes significantly correlated with both PD-1 and PD-L1. Blue indicates genes significantly correlated only with PD-1. Green indicates genes significantly correlated only with PD-L1. Gray color indicates genes with no correlation with PD-1 or PD-L1. The black triangle indicates the correlation between PD-1 and PD-L1. Significant correlation means |Cor.| > 0.25 and *p* < 0.05. Cor. indicates Pearson's correlation coefficient.

It has been confirmed that the downregulation of IL-17A and IL-17F by the estrogen receptor affects the expression of genes encoding downstream products of IL-17A/IL-17F signaling transduction. Thus, we explored whether IL-17 signaling pathway-related genes were coexpressed with PD-1/PD-L1 in breast cancer, especially in ER-negative samples. Three cohorts were used for the correlation analysis of the expression of PD-1/PD-L1 vs. that of 94 genes. There were twenty genes in the cohort that were present all samples; 20 genes in the cohort of ER-negative samples and 18 genes in the cohort of basal-like samples were observed to be positively correlated with PD-1 and/or PD-L1. No IL-17 signaling pathway-related genes were found to be significantly negatively correlated with PD-1 or PD-L1 (Figure 6). This proves that an enhanced IL-17 signaling response increases the expression level of PD-1/PD-L1 in breast cancer. IFNγ (*IFNG*), CXCL10, CCL2, and GM-CSF (*CSF2*) were confirmed to have a stronger correlation with PD-1/PD-L1 in the basal-like cohort. It was noticeable that IFNγ showed a remarkable positive correlation with both PD-1 and PD-L1 in each cohort. In addition, several regulatory genes in the IL-17 signaling pathway, such as *TNFAIP3, TRADD*, and *IKBKE*, were also observed to be correlated with PD-1 and/or PD-L1 in one or more cohorts of breast cancer samples.

### Expression of Genes Related to the IL-17 Signaling Pathway Is Positively Correlated With the Infiltration of CD8^+^ T Cells and the Immune Microenvironment in Breast Cancer

We further studied the effect of the IL-17 signaling pathway on the immune microenvironment of breast cancer. IL-17A and IL-17F play a role in inducing the expression of chemokines and cytokines, recruiting immune cells, and promoting inflammation. Therefore, it is expected that genes related to the IL-17A/IL-17F signaling pathway increases immune infiltration in breast cancer tissues, especially genes that encode downstream products of IL-17A/IL-17F signaling transduction. The level of CD8^+^ T cell infiltration was the focus because of its relationship with the effectiveness of immunotherapy and the prediction of prognosis. It was confirmed that the expression of 60 genes among 91 IL-17 signaling pathway-related genes for which expression data were available increased the infiltration of CD8^+^ T cells in breast cancer, while the expression of eight genes (including *IL25*) showed a weakly negative correlation with CD8^+^ T cell infiltration. For basal-like samples, most IL-17 signaling pathway-related genes showed no significant correlation with CD8^+^ T cell infiltration, which was probably due to the small cohort size. Only 30 genes were positively correlated with CD8^+^ T cells, and two genes were negatively correlated with the basal-like cohort. Significant correlations between genes and CD8^+^ T cell infiltration in the two cohorts are shown in Table 1 and are sorted by the correlation coefficient. The genes with a significant positive correlation are almost identical between the two cohorts. TRAF6 mediates the intracellular transduction of IL-17 signaling (29). TNFAIP3 (30) and USP25 (31) regulate IL-17A/IL-17F signaling by inhibiting TRAF6. MAPK14, MAPK1, and TAB2 are involved in IL-17-dependent activation of MAPK or NF-κB. The six genes mentioned above were observed to upregulate CD8^+^ T cell infiltration. On the other hand, IFNγ, CXCL10, and CCL2 have been shown to be cytokines that remarkably elevate CD8^+^ T cell infiltration.

**Table 1:** IL-17 signaling pathway-related genes were significantly correlated with the infiltration of CD8^+^ T cells in breast cancer.

**Infiltration of CD8^**+**^ T cells**	**TCGA-BRCA**		**TCGA-BRCA basal-like**	
	**Cor**.	***p***	**Cor**.	***p***
**Genes that participated in transduction and the regulation of IL-17**				
**signaling**				
*TNFAIP3*	0.5675	^***^	0.3069	^***^
*MAPK14*	0.4306	^***^	0.3049	^***^
*MAPK1*	0.4152	^***^		
*TRAF6*	0.4032	^***^	0.2815	^**^
*TAB2*	0.3995	^***^	0.2750	^**^
*USP25*	0.3719	^***^		
*CHUK*	0.3630	^***^	0.3061	^***^
*TAB3*	0.3277	^***^	0.3700	^***^
*CASP8*	0.3588	^***^	0.3592	^***^
*TBK1*	0.3553	^***^	0.3120	^***^
*CASP3*	0.3519	^***^	0.2738	^**^
*MAP3K7*	0.3374	^***^	0.2971	^***^
*MAPK6*	0.3327	^***^		
*IL17RA*	0.3308	^***^		
*NFKB1*	0.3254	^***^	0.2668	^**^
*MAPK8*	0.3003	^***^		
*GSK3B*	0.2889	^***^		
*TRAF3*	0.2688	^***^		
*MAPK9*			0.3195	^***^
*HSP90B1*			0.2542	^**^
**Genes encoding downstream products of IL-17 signaling transduction**				
*IFNG*	0.4691	^***^	0.5530	^***^
*CXCL10*	0.4441	^***^	0.4145	^***^
*CCL2*	0.3790	^***^	0.3905	^***^
*IL6*	0.2579	^***^	0.3425	^***^
*PTGS2*	0.3163	^***^		
*CCL11*	0.3116	^***^		
*IL1B*	0.2652	^***^	0.2700	^**^
*MMP3*	0.2669	^***^		
*CSF2*			0.3306	^***^
*IL13*			0.2665	^**^

*|Cor.| > 0.25*,

** *p < 0.01*,

*** *p < 0.001*.

**Figure 7:**
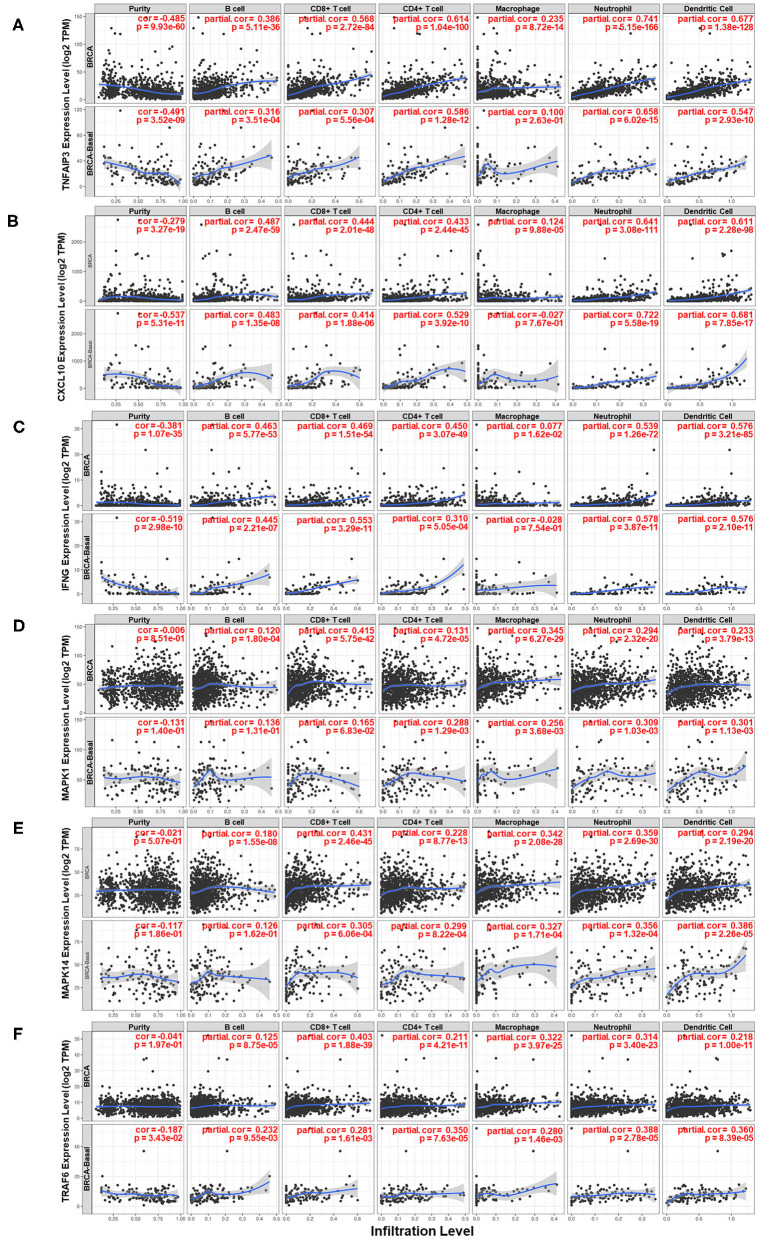
Analysis of the associations between six key genes and immune infiltration in breast cancer. Correlations of the expression of **(A)** *TNFAIP3*, **(B)** *CXCL10*, **(C)** *IFNG*, **(D)** *MAPK1*, **(E)** *MAPK14*, and **(F)** *TRAF6* with the immune microenvironment and tumor purity. The panels in the upper row of each figure are based on the total samples in the TCGA Breast Cancer dataset, and the panels in the bottom row of each figure are based on samples in the basal-like subgroup of the TCGA Breast Cancer dataset.

Moreover, the relationship between several genes and the immune microenvironment of breast cancer tissue was studied. The association of the expression levels of *TNFAIP3, CXCL10, IFNG, MAPK1, MAPK14*, and *TRAF6* with the infiltration levels of several major immune cell subsets in breast cancer is shown in Figure 7. The expression level of genes compared to tumor purity is displayed in the leftmost panel of each figure, which helps to clarify the cell source of gene expression. *TNFAIP3, CXCL10*, and *IFNG* exhibited high expression levels in cells derived from the microenvironment, although it seemed that only a partial contribution was associated with the IL-17 signaling pathway. *TNFAIP3* was strongly associated with the infiltration of all types of immune cells in breast cancer, and *CXCL10* and *IFNG* behaved similarly in all immune cell types except macrophages. Two members of the MAPK family, *MAPK1* and *MAPK14*, showed a similar pattern that they mainly influenced CD8^+^ T cells, macrophages, neutrophils, and dendritic cells. *TRAF6* was strongly correlated with CD8^+^ T cells but intermediately correlated with other cell subsets of the immune microenvironment.

## Discussion

**Figure 8:**
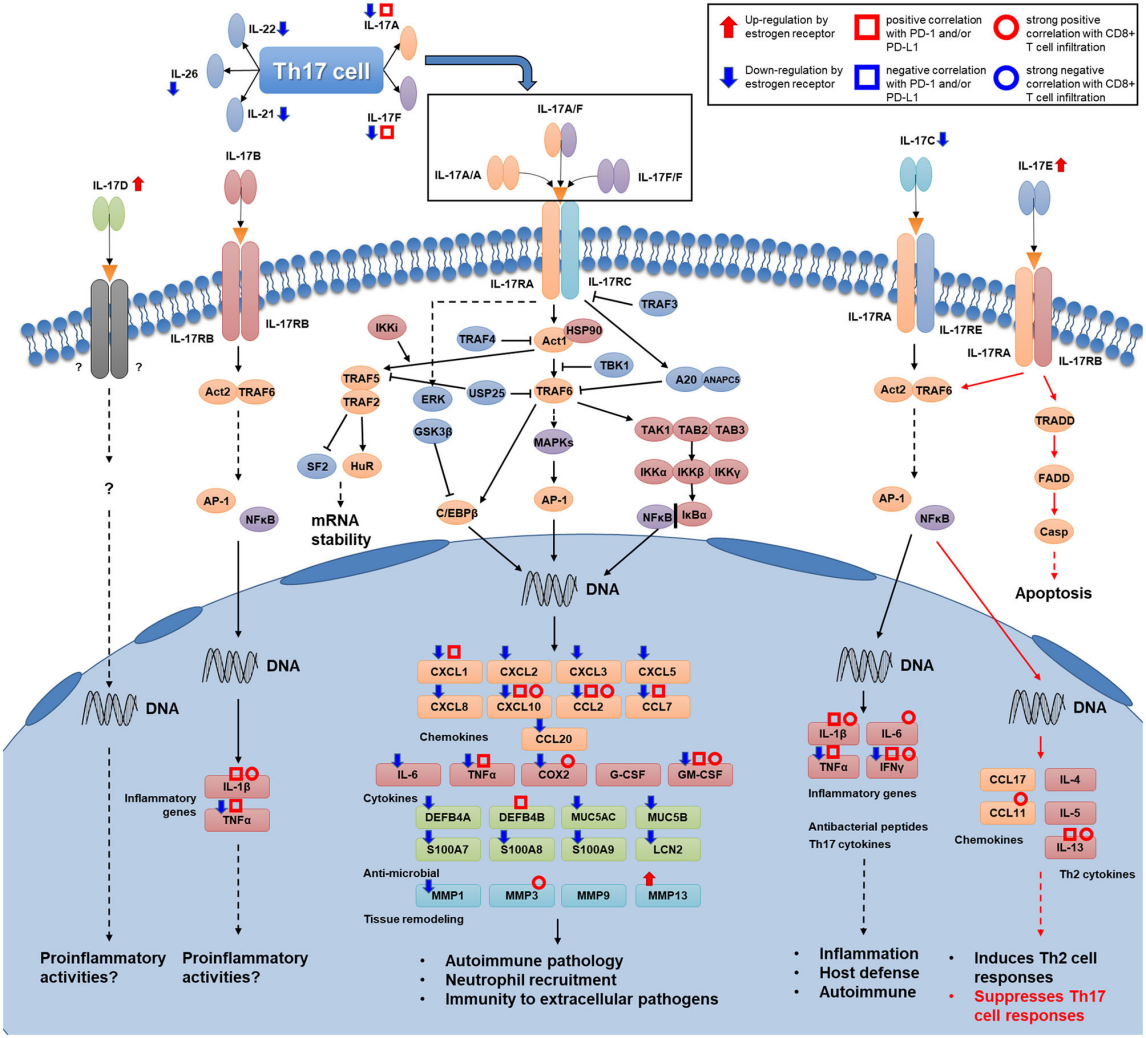
Map of Th17 cell signature cytokines and the IL-17 signaling transduction pathway. Several kinds of associations that were proven in this study are shown for several genes (Th17 cytokine genes, IL-17 family cytokine genes, and genes encoding downstream products of IL-17 signaling transduction) in this figure. Associations of genes that participate in intracellular transduction and the regulation of IL-17 signaling are not shown in the figure.

The purpose of our study was to investigate how IL-17 signaling mediates the decrease in PD-1/PD-L1 expression and CD8^+^ T cell infiltration due to high ER expression levels in breast cancer. Comprehensive analysis of cytokines of the IL-17 family suggested that high levels of ER decrease the expression of IL-17A, IL-17F, and IL-17C while increasing the expression of IL-17E (*IL25*). Since IL-17A, IL-17F, and IL-17C are initiators of IL-17 signal transduction, whereas IL-17E suppresses Th17 cell responses, ER downregulates IL-17 signal transduction in breast cancer by differentially regulating the expression of IL-17 cytokines. Since the Th17 cell subset is a major cellular source of IL-17A and IL-17F, we hypothesized that the downregulation of these two cytokines was related to the number of Th17 cells in tumor tissue. Five signature cytokines of the Th17 cell subset were downregulated in breast cancer with a high level of ER expression, indicating the poor infiltration of Th17 cells in this subtype. This finding reveals a possible reason for the decreased expression of IL-17A and IL-17F. On the other hand, the majority of genes encoding downstream products of IL-17A and IL-17F signaling transduction had increased expression levels in ER-negative and basal-like breast cancer. This suggests that a high level of ER expression weakens the response to IL-17A and IL-17F signaling. Next, an analysis established the associations between the high expression of PD-1/PD-L1 and ER-negative status, IL-17A-positive status, and IL-17F-positive status and proved that a series of IL-17 signaling pathway-related genes showed a positive correlation with PD-1 and/or PD-L1 expression levels. This indicates that the downregulation of IL-17A and IL-17F signal transduction is a possible mechanism involved in the decrease in PD-1/PD-L1 expression in breast cancer with a high level of ER expression. Furthermore, the analysis verified our hypothesis that the enhanced IL-17 signaling response increases CD8^+^ T cell infiltration in breast cancer, which provides a possible explanation for the high CD8^+^ T cell infiltration level in ER-negative breast cancer or TNBC. Finally, a brief depiction of how IL-17 signaling stimulates various cytokines and chemokines to influence the immune microenvironment in breast cancer was made. Figure 8 shows the IL-17 signaling pathway and the signature cytokines of Th17 cells. The associations between several of the IL-17 signaling pathway-related genes and ER status, PD-1/PD-L1 expression levels, and CD8^+^ T cell infiltration level are highlighted.

According to this study, we propose a possible mechanism by which a highlevel of ER reduces Th17 cell infiltration in tumor tissues, thereby decreasing the levels of IL-17A and IL-17F, weakening the intensity of IL-17A and IL-17F signal transduction, and finally downregulating the expression level of PD-1/PD-1 and the infiltration level of CD8^+^ T cells in breast cancer. This mechanism explains why increased levels of IL-17A (7–10) and PD-L1 (12, 13) were discovered in breast cancer with a low level of ER expression. Many studies have provided evidence to support this mechanism. First, it has been discovered in several pathological processes that estrogen deficiency leads to an increase in Th17 cell infiltration and the upregulation of IL-17 signal transduction. Tyagi et al. (32) observed an increase in Th17 cell differentiation and IL-17 levels in ovariectomized mice, and these effects were reversed after estrogen supplementation. A deficiency in estrogen induced the upregulation of the IL-17 effector ACT1, which promotes bone resorption in postmenopausal osteoporosis (33). In experimental arthritis, estrogen treatment reduced the number of IL-17-positive γδT cells in joints while increasing the number in draining lymph nodes (34). A previous study confirmed that the level of Th17 cell infiltration is higher in breast cancer tissue than that in normal breast tissue, and it is positively associated with the expression levels of IL-17, IL-1β, and IL-6 (35). In addition, the association between IL-17 and PD-1/PD-L1 has been observed in several kinds of carcinoma. Aotsuka et al. (36) demonstrated that IL-17A induced the expression of PD-L1 in ovarian cancer cell lines. Wang et al. (37) observed that IL-17A upregulated PD-L1 protein levels in human prostate cancer and human colon cancer cell lines. Yang et al. (38) studied interleukin-17 receptor C (*Il17rc*), a subunit of the IL-17A receptor, in a mouse prostate tumor model and found significantly higher levels of PD-1 and PD-L1 in *Il17rc* wild-type tumors than in *Il17rc* knockdown tumors. Finally, Amicarella et al. (39) proved that colorectal cancer-derived Th17 cells promote the recruitment of CD8^+^ T cells and neutrophils into tumor tissue via the secretion of IL-17, which suggests the role of the IL-17 signaling response in changing the immune microenvironment of tumors. In summary, the results of our analysis suggested that the mechanism described above also plays a role in breast cancer.

In addition to those genes that encode downstream products of IL-17A/IL-17F signaling transduction, the expression levels of *IFNG, FOS, FOSB1*, and *FOSL1* were also found to be associated with ER status in breast cancer. Downregulation of IL-17C and its downstream cytokine IFNγ in breast cancer with a high level of ER expression indicates that IL-17C signaling transduction may also be associated with the regulation of ER. On the other hand, FOS, FOSB1, and FOSL1 participate in the formation of the AP-1 transcriptional complex. A previous study suggested an increase in the abundance of AP-1 and an increase in the levels of IFNγ in ER-negative breast cancer, whereas AP-1 levels were not correlated with PR status, tumor grade, tumor size, or lymph node status (40). In addition, moderate or strong *FOSB* expression has been confirmed to be correlated with ER-positive status and PR-positive status in breast cancer tissue and cell lines (41), which is consistent with our findings. *IFNG, CXCL10, CCL2*, and *TNFAIP3* were identified as hub genes involved in increasing the expression of PD-1/PD-L1 and elevating CD8^+^ T cell infiltration by our analysis. Previous studies have suggested that effector T cell-derived IFNγ contributes to high levels of PD-1 expression in the tumor microenvironment (42); CCL2 attracts monocytes, increases the number of tumor-associated macrophages and promotes angiogenesis in breast cancer (43). Overexpression of CXCL10 is considered to be a favorable prognostic factor in TNBC (44), and high levels of TNFAIP3 expression are correlated with poor overall survival in HER2^+^ breast cancer and TNBC (45). We observed a close relationship between these hub genes and neutrophil infiltration in breast cancer tissue, suggesting that IL-17 cytokines attract and activate neutrophils by stimulating the production of neutrophilic chemokines. It is well-known that tumor-associated neutrophils can also stimulate the production and release of these cytokines, such as IFN-γ and CCL2 (46). However, further investigation is required to clarify the contribution to these effects of IL-17 signaling transduction because these cytokines are involved in many biological processes and signaling transduction pathways.

Our research is based on mRNA sequencing data from the TCGA Breast Cancer dataset. TCGA Breast Cancer is the largest breast cancer public database that, to the best of our knowledge, provides complete expression data for IL-17 signaling pathway-related genes. To verify our conclusions, further analysis based on multiple databases and more data types (DNA copy number variation, DNA methylation, etc.) is needed, which is the main limitation of this work. Qualitative analyses were used for the expression data for several cytokines because of their low expression levels in breast cancer tissue. To obtain more convincing quantitative results, appropriate *in vitro* experiments may be required. In addition, it is expected that the five signature cytokines of Th17 cells are negatively correlated with tumor purity since they are derived from cells in the microenvironment (mainly from the Th17 cell subset). However, we did not observe a significant correlation between the cytokines IL-17F and IL-22 and tumor purity, which may also be due to the low expression level of these cytokines. In addition, we are able to illustrate the association between IL-17 signaling pathway-related genes and ER status, the PD-1/PD-L1 level, or immune infiltration in breast cancer. However, it is difficult to determine whether the association can be attributed to IL-17 signal transduction from the perspective of a single gene. Therefore, further analysis based on these hub genes could be carried out in the future.

In conclusion, our study demonstrated that a high level of estrogen receptor expression decreases PD-1/PD-L1 expression and CD8^+^ T cell infiltration by suppressing Th17 cell infiltration and IL-17 signaling transduction in breast cancer. This finding provides new evidence to explain the role of the IL-17 family of cytokines in breast cancer.

## Data Availability Statement

All datasets generated for this study are included in the article/Supplementary Material.

## Author Contributions

Study design: CS and WH. Funding acquisition: HP and WH. Data collection: CS. Data analysis: CS and XY. Figures and tables drawing: CS. Original draft writing: CS and WH. Manuscript review: HP and WH. Reading, comments, and approval of final manuscript: All authors.

## Conflict of Interest

The authors declare that the research was conducted in the absence of any commercial or financial relationships that could be construed as a potential conflict of interest.

## Footnotes

^1^http://xena.ucsc.edu/

^2^https://cistrome.shinyapps.io/timer/

^3^http://www.genome.jp/kegg
